# Indirect Reciprocity, Resource Sharing, and Environmental Risk: Evidence from Field Experiments in Siberia

**DOI:** 10.1371/journal.pone.0158940

**Published:** 2016-07-21

**Authors:** E. Lance Howe, James J. Murphy, Drew Gerkey, Colin Thor West

**Affiliations:** 1 Department of Economics and Public Policy, University of Alaska Anchorage, Anchorage, Alaska, United States of America; 2 Institute of State Economy, Nankai University, Tianjin, China; 3 Economic Science Institute, Chapman University, Orange, California, United States of America; 4 Department of Anthropology, School of Language, Culture & Society, Oregon State University, Corvallis, Oregon, United States of America; 5 Department of Anthropology, University of North Carolina, Chapel Hill, North Carolina, United States of America; Goethe-Universitat Frankfurt am Main, GERMANY

## Abstract

Integrating information from existing research, qualitative ethnographic interviews, and participant observation, we designed a field experiment that introduces idiosyncratic environmental risk and a voluntary sharing decision into a standard public goods game. Conducted with subsistence resource users in rural villages on the Kamchatka Peninsula in Northeast Siberia, we find evidence consistent with a model of indirect reciprocity and local social norms of helping the needy. When participants are allowed to develop reputations in the experiments, as is the case in most small-scale societies, we find that sharing is increasingly directed toward individuals experiencing hardship, good reputations increase aid, and the pooling of resources through voluntary sharing becomes more effective. We also find high levels of voluntary sharing without a strong commitment device; however, this form of cooperation does not increase contributions to the public good. Our results are consistent with previous experiments and theoretical models, suggesting strategic risks tied to rewards, punishments, and reputations are important. However, unlike studies that focus solely on strategic risks, we find the effects of rewards, punishments, and reputations are altered by the presence of environmental factors. Unexpected changes in resource abundance increase interdependence and may alter the costs and benefits of cooperation, relative to defection. We suggest environmental factors that increase interdependence are critically important to consider when developing and testing theories of cooperation

## Introduction

Survival of small-scale subsistence-dependent communities often depends upon whether individuals can successfully overcome the collective action problem (also known as a social dilemma). In these situations, the group is collectively better off when all individuals cooperate. However, individuals have an incentive to free-ride, or defect on the cooperation of others. The standard prisoner’s dilemma is a special case of this class of problems. Research on cooperation and collective action often focuses primarily on strategic risks—the costs and benefits of cooperating or defecting—and associated free-riding behavior [1,2]. However, cooperation often occurs in multiple domains. In addition to contributing to the public good or harvesting from a shared resource, what we refer to as the “production domain,” cooperation can also emerge in other domains, such as punishing defectors [3–6], rewarding cooperators [7–9] or sharing with those who experience a hardship [10]. These domains often interact which reflects the fact that benefits of cooperation can extend beyond a single period, domain, or state of nature [11]. Cooperation may be preferred to non-cooperation precisely because future states of nature are uncertain in one or more linked domains [12]. As such, environmental risk—defined as the spatial and temporal fluctuations in biotic and abiotic components of the environment that affect access to resources, health, and other measures of human well-being—could increase interdependence. Under such conditions, long-term success may depend upon cooperation in multiple domains.

Idiosyncratic environmental risk varies across individuals within a community, creates uncertainty about future payoffs and can threaten an individual’s survival. Individual harvesting success may be stochastic, harvested resources may spoil, animals may destroy stored food, or an injury may prevent the individual from participating in collective action. In subsistence communities, when an individual experiences a hardship, or a “shock,” his or her well-being depends upon the generosity of others. This can manifest itself through indirect reciprocity—helping those who help others. Although helping others may be costly, in doing so the individual forms a reputation for cooperative behavior that may yield benefits in a future time of need [13]. Decisions about sharing subsistence resources may therefore depend upon the recipient’s reputation for cooperating in other domains. In an environment with both production and sharing domains, indirect reciprocity is defined as the sharing given to an individual that is conditioned on the observed cooperation of that individual in both domains [14–16]. As a result, although environmental risk can increase variation in the production domain and reduce harvests for some, sharing among individuals and households can compensate for these short-term deficits, linking the strategic dynamics of cooperation across the two domains [17–20].

In this paper we present results from a framed public goods experiment, conducted in subsistence-dependent communities in Siberia. The experiments were designed to test how idiosyncratic environmental risk interacts with strategic risk to affect cooperation within and between the production and sharing domains. Consistent with a model of indirect reciprocity, our results indicate that decisions in the sharing domain were affected by reputations for cooperation in the production domain. We also find evidence for risk-pooling (i.e., individuals shared more with those in need). Further, when reputations for cooperation extended across multiple rounds, the aid provided to cooperators increased substantially and risk-pooling became more effective. These results highlight the importance of local social norms which emphasize resource sharing and helping the needy [21,22]. However, the rewards from sharing in our experiments were insufficient to improve cooperation in the production domain. In addition, we find cooperation in the social dilemma was unaffected by unavoidable individual risk, consistent with theoretical predictions.

## Environmental Risk and Cooperation

Because environmental risk introduces variability in resource acquisition, it can be difficult or impossible for a solitary individual to consistently acquire sufficient resources to survive. As a result, environmental risks can affect the relative viability of independent versus cooperative behavior. Previous research has shown that environmental risk affects cooperation over rivalrous goods in small-scale, resource-dependent communities [23–26]. In theoretical studies, whether environmental risk or uncertainty will have a positive or a negative effect on cooperation in social dilemmas can depend on whether players are working to provide a public good or to reduce a public bad [27]. Experimental studies generally find that increasing the variability of returns to either collective action or to independent action reduces cooperation in the riskier domain. For instance, when risk is associated with collective action, participants increase extraction requests and overharvest in the case of a common pool resource or under-contribute in the case of a public good [28–31].

Although environmental risks have received relatively less attention in research on cooperation and collective action compared to strategic risks, there is a large literature within anthropology [32,33] and economics [34–36] that has explored the theoretical and empirical dimensions of risk and the mechanisms used to pool resources at the community level to offset negative shocks to individual or household income or food consumption–a.k.a. “risk-pooling.” Smith [37] suggests risk-pooling is likely to occur when an individual’s success in resource acquisition exhibits stochastic variation that is asynchronous among individuals, creating opportunities for individuals to reduce environmental risk by sharing resources. Related economic studies have identified the use of non-market mechanisms—including informal loans, remittances, and social networks—to pool risk and minimize the negative effects of consumption variability [19,38,39].

When individual resource production declines due to unexpected changes in abundance or success, deficits can be reduced by pooling resources, which enhances odds of survival. This form of cooperation entails strategic risk in both the production and sharing domains. In the production domain, individuals who produce less due to lack of effort avoid the costs associated with production, but still stand to benefit from sharing. In the sharing domain, individuals who receive aid during times of need but do not provide aid to others avoid the costs associated with sharing. In both cases, the decision to produce or share is strategic in that outcomes depend on the decisions of other players. The structure of the social dilemma provides each player with an incentive to make decisions that increase individual earnings at the expense of collective outcomes. Theoretical models of risk-pooling have shown a strong commitment device (a mechanism in which the long-term benefits of participation exceed the short-term gains of defection) can facilitate effective risk-pooling [40]. When risk-pooling is effective, individual consumption (or income) is largely unaffected by unexpected changes in resource abundance or success [35].

Experimental research echoes the results of these models. Studies have explored commitment in the context of endogenous group formation [41]. For example, Barr and Genicot [42] found individuals were most likely to form risk-pooling groups in the presence of a strong, exogenously enforced, commitment device. When earnings were pooled and evenly distributed, or a fine was imposed by the experimenter on any player for under-contributing to the group activity or for withholding sharing, strategic risks of cooperation were reduced and risk-pooling became more effective. In the absence of an exogenously enforced commitment device, risk-pooling can still arise through direct reciprocity. When interactions are repeated and partners have opportunities to monitor reputations, reciprocity can enhance risk-pooling as partners give or withhold resources, depending on a partner’s previous actions [43].

Our study complements previous research by investigating factors that enhance risk-pooling in the absence of exogenously enforced commitment devices and direct reciprocity. Specifically, we combined environmental factors that increase interdependence and encouraged risk-pooling with factors that amplified strategic risks of defection, including reputations, rewards, and punishments. We utilized methodological tools from anthropology and economics to design a series of field experiments involving 136 participants from three villages located on the Kamchatka Peninsula in Northeast Siberia. Prior to the experiments, we conducted qualitative ethnographic interviews and participant observation to identify the particular strategic and environmental risks that people in Kamchatka face. These insights informed the design of our experiments. People living in Kamchatka—particularly indigenous Koryaks, Chukchis, Evens, Itlemens—have developed cultural norms and institutions that foster cooperation and resilience despite challenging social and ecological conditions. Traditional subsistence activities like reindeer herding, salmon fishing, and foraging are inherently cooperative, and unfold in arctic and sub-arctic environments characterized by high degrees of seasonal and annual variation in weather and resource availability. Moreover, subsistence strategies—and the cultural norms and institutions in which they are embedded—have helped people adapt to economic and political upheavals during Soviet era collectivization and post-Soviet privatization [44]. This unique combination of social and ecological uncertainty has fostered cultural norms and values that encourage cooperation and risk-pooling. As one person explained, “If someone has misfortunes you try to support them so the person isn’t let loose. That is, reciprocity here is a very good, necessary thing.” Another person put it more bluntly, “Because in the North, I say, a loner doesn’t survive” [45].

## Research Design

Our field experiments were conducted in three small communities in the Karaginskii Region of Kamchatka over a four day period in each community during Spring 2011. This is a large, remote region (40,600 km^2^) with a small population (4,824 people) that is dependent upon harvesting local resources for subsistence. Approximately 85% of experiment participants were indigenous and had lived in the area for most of their lives. Table 1 provides some descriptive statistics for our experiment participants.

**Table 1:** Participant characteristics.

Number of participants	136
Percent female	66.1%
Mean age (years)	36.8
Percent indigenous	85.2%
Mean years of education	10.6

Participants were recruited through bulletin board announcements, door-to-door visits, and by a local community coordinator. Experiments were conducted in Russian and all supporting materials were presented in Russian. Participants read a consent form prior to the start of the experiment and provided verbal affirmation of informed consent prior to participation. Signatures were not collected since our study was determined to be of minimal risk, participants experienced risk similar to that encountered in everyday life, and signatures would have unnecessarily linked subjects to the study. Investigator contact information was provided to participants and left with village mayors and community coordinators. Researchers returned to the communities two years later to report related research findings to participants and community members. Our study and consent procedures were approved by the University of Alaska Anchorage (UAA) Institutional Review Board (project id #216266). The protocol was pre-tested with native Russian-speaking students at UAA. Instructions were read aloud and accompanied by PowerPoint slides projected onto a screen. Instructions in English and Russian, field protocols, and an image of information displayed to participants, can be found in the Supporting Information.

Each session lasted approximately three hours, during which participants played a modified version of a linear public goods game followed by a short demographic survey and a dictator game (which is not discussed in this paper). Experiments were hand-run, with the aid of a single laptop computer and a projector. For each round, decisions were written on slips of paper, collected by one of the experimenters, and entered into a spreadsheet. Results were projected onto the screen, and participants wrote the outcomes on a record sheet. Once this process was completed, another round followed.

Participants were randomly assigned to one of two five-person groups. In 4 of the sessions there was one 5 person group and one 4 person group. In the remaining 10 sessions there were 2 five person groups for a total of 136 participants. In all treatments, individuals were identified by a letter known only by the individual and the experimenter. Thus, participants knew the composition of each group, but there was no way for other group members to link an individual to his or her decisions. Moreover, with one exception (described later), each individual’s letter randomly varied every round. This method eliminated the possibility of using information about a particular group member’s actions in prior rounds and prevented individuals from developing reputations. Participants were paid in cash, with average earnings of 610 rubles (about $22 US dollars at the time), equivalent to a typical daily wage. In addition, all participants received a 200 ruble show-up payment.

The modified public goods game was framed as team production [46,47], and consisted of five treatments. For the first five rounds, all players participated in the Baseline Treatment, a standard linear public goods game. Each round, every individual started with an initial endowment of 50 “hours” which had to be allocated between an individual and a group subsistence activity. The activity was framed as “fishing, hunting, or collecting mushrooms and berries…” where “sometimes you do these activities on your own” (the individual production activity) but “sometimes you do them with other people” (the group production activity). Each hour allocated to the individual activity yielded a private return of 10 rubles. Time allocated to the group activity yielded 20 rubles per hour, because “people often get more done when working together.” Returns from the group activity were divided equally among all group members, regardless of the time allocated. At the end of each round, the allocation decision and earnings of each group member were publicly revealed, identified only by each individual’s letter.

Over the next eight rounds of the game, subjects participated in one of four sharing treatments. Treatments varied in terms of the presence of environmental risk and the strategic risks of cooperation as determined by the information available to participants when making decisions. In all treatments, participants first made the same time allocation decision as in the Baseline Treatment. After the decisions were made, some information was revealed (which varied by treatment), then participants were given the opportunity to share rubles with other group members. The instructions emphasized the voluntary nature of sharing and used the Russian verb *podelit’sia* (“to share”). There was no restriction on the number of fellow group members with whom an individual could share. To avoid sharing commitments in excess of an individual’s earnings, the total amount shared by an individual was limited to 250 rubles. Table 2 summarizes the experimental design and key information revealed for each treatment.

**Table 2:** Experimental Design.

		**Risk and Sharing Treatment**		**Information Revealed Prior to Sharing Decision**	
**Treatment**	**N**	**Idiosyncratic Risk**	**Voluntary Sharing**	**Current round investment decisions of each player**	**All previous investment & sharing decisions for each player**
Baseline	136	no	no	—	—
T1: Reward	40	no	yes	yes	no
T2: Risk	29	yes	yes	no	no
T3: Risk/Reward	38	yes	yes	yes	no
T4: Risk/Reward/ Reputation	29	yes	yes	yes	yes

### T1. Reward Treatment

The first treatment was identical to the Baseline except that after time allocation decisions were completed and publicly revealed, participants made a sharing decision. Because individual time allocation decisions were common knowledge, participants could use sharing as a mechanism to reward others for contributing to the group activity in the current period or to indirectly punish non-cooperators by withholding sharing, increasing the cost of defection relative to the Baseline Treatment. Because sharing involved transfers between players, it had no impact on group earnings. After sharing decisions were collected, the amounts shared and received were revealed to the group. In all treatments with sharing (i.e., the four treatments in rounds 6–13), only aggregate sharing outcomes were revealed, so the amount transferred between two particular players was not disclosed. This treatment is similar to the Reward Treatment in Sefton, Shupp and Walker [48].

### T2. Risk Treatment

The second treatment introduced idiosyncratic environmental risk. After the time allocation decisions were made, but before they were revealed, one individual from each group was randomly selected by the roll of a die to incur a “shock” or hardship which was described as “not catching any fish, getting sick, or having all the food you’ve gathered spoil.” The individual who incurred the shock lost all earnings from both the group and individual activities. Only the amount received from voluntary sharing by others determined the individual’s earnings for that round. This shock is consistent with our ethnographic research in Kamchatka, which indicates households frequently experience idiosyncratic reductions in subsistence harvests due to illness or injury, lose harvested resources through spoilage, and experience fluctuations in income due to economic uncertainty and wide-spread unemployment. In each treatment with environmental risk, the letter of the individual incurring the shock was announced to the group prior to the sharing decision. After the sharing decisions were collected, both the time allocation and sharing decisions of all group members were revealed.

### T3. Risk/Reward Treatment

The third treatment was identical to the Risk Treatment, except that prior to the sharing decision, both the letter of the individual who received the shock and the allocation decisions of all group members were revealed. This allowed sharing to be based on whether an individual was shocked and/or the individual’s time allocation in the current period. After the sharing decisions were collected, the individual sharing and time allocation decisions were revealed to the group.

### T4. Risk/Reward/Reputation Treatment

The final treatment (which for conciseness, we will refer to as the Reputation Treatment) followed the same rules as the Risk/Reward Treatment, but the individual player letters were constant across rounds. Holding player letters constant created an opportunity for participants to develop a reputation for cooperative behavior not only in the production domain, but also the sharing domain. This allowed other group members to condition sharing on these reputations. The Reputation Treatment brings the experiment closer to naturally occurring contexts of cooperation in small-scale societies, where individuals have access to and utilize reputations through repeated interactions.

Individual cash earnings were determined by a single round that was randomly selected by a die roll at the end of the experiment [10,43]. Selecting a single round eliminated the possibility for participants to pool earnings over time, which would have been analogous to individually insuring against shocks. Our design choice parallels field conditions in Kamchatka where there is substantial seasonal variation in weather and resource availability and it is difficult for individuals to endure deficits in subsistence harvests without aid from others in the community.

### Related Studies and Hypotheses

The design of our experiment was most similar to a computerized laboratory experiment by Cherry, Howe, and Murphy [10] but differed in terms of the framing, the source of the shock, the nature of sharing, and the amount of information revealed. They found strong evidence for risk-pooling without a commitment device. In contrast, our design introduced unavoidable idiosyncratic risk and allowed us to test the effect of reputations on sharing and cooperation decisions.

In each of our treatments, the static Nash equilibrium allocations to the group activity and to sharing were both zero. Further, because direct reciprocity was not possible in our experiments, sharing arrangements were not self-enforcing. The expected future individual gain from cooperating by sharing did not exceed the current benefit of defecting. Essential features of this experiment have been modeled by Nowak and Sigmund [15] who explored cooperation via indirect reciprocity. A growing number of experimental studies provide support for the importance of reputation and the role of indirect reciprocity in cooperation and collective action [8,14,15,49,50]. In the context of two linked cooperative domains, Panchanathan & Boyd [16] suggested indirect reciprocity depends on two conditions: 1) reputations formed by actions in the first domain increase benefits received in the second domain and 2) the benefits of a good reputation in the second domain exceed the costs of cooperation in the first domain. We investigate how environmental risk affects these strategic dynamics of reputation and indirect reciprocity.

By comparing decisions across the treatments, we can test the extent to which time allocated to the group activity and sharing decisions are interlinked and how they respond to strategic and environmental risk. Based on the Panchanathan & Boyd [16] model of indirect reciprocity, we hypothesize that sharing decisions will be conditioned on observable behavior, and that people who exhibit more cooperation in the observed domains will receive more support. This implies that in the Reward Treatment, participants will use sharing to reward cooperation and will withholding sharing from non-cooperators (H1). In the Risk Treatment, those experiencing a hardship will receive additional support, but it will be independent of time allocation and sharing decisions because these are unobservable (H2). In the Risk/Reward Treatment, we expect that sharing will be directed towards the individual who was shocked and sharing will increase with the shocked individual’s group allocation decision in the current round (H3). In the Reputation Treatment, the amount shared with a shocked individual should increase with both his or her allocation decision in the current period and his or her sharing decision in the previous period (H4). Hypotheses 3 and 4 focus on the sharing domain in the presence of idiosyncratic environmental risk, specifically whether the amount received from sharing is conditioned on both whether an individual experiences a hardship and observed cooperative behavior. Assuming these two hypotheses are supported, Hypothesis 5 tests whether there is an interdependence between the sharing and production domains. If we observe that sharing is directed toward those who incur a shock and is based on a recipient’s contributions to the group activity, then we expect the amount of time allocated to the group activity in the production domain will be greater in the final two treatments compared to the Risk treatment (T2), since sharing received in these treatments would provide an additional benefit from investing in the group activity (H5). In the presence of idiosyncratic environmental risk, when an individual’s behavior is observable, there could be an incentive to behave more cooperatively (relative to the Risk treatment in which cooperation is not observed) because doing so is likely to yield additional support should one experience a hardship—essentially the additional cooperation in the production domain could be a form of insurance against an adverse outcome.

## Results

**Fig 1:**
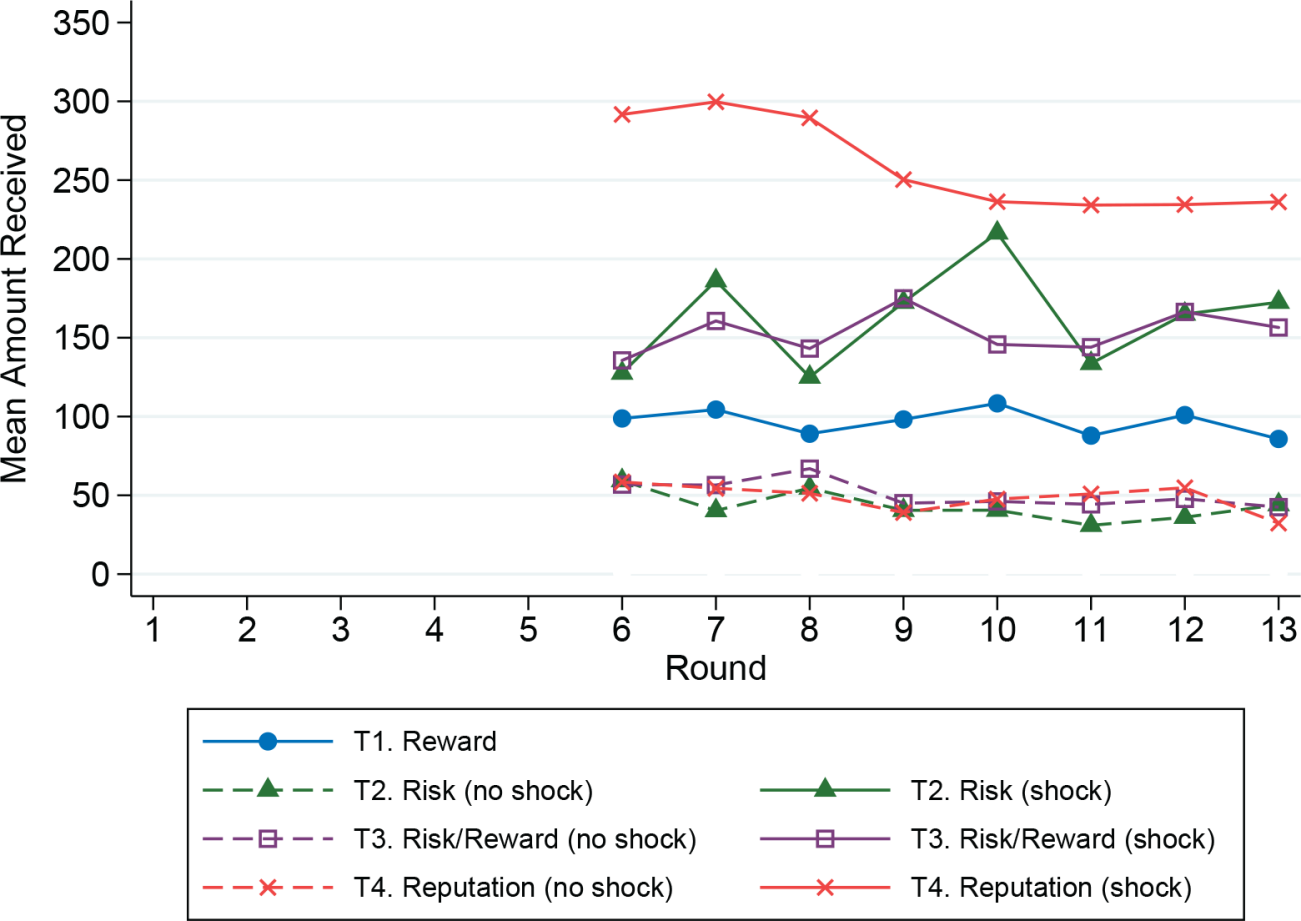
Mean amount received from sharing.

**Fig 2:**
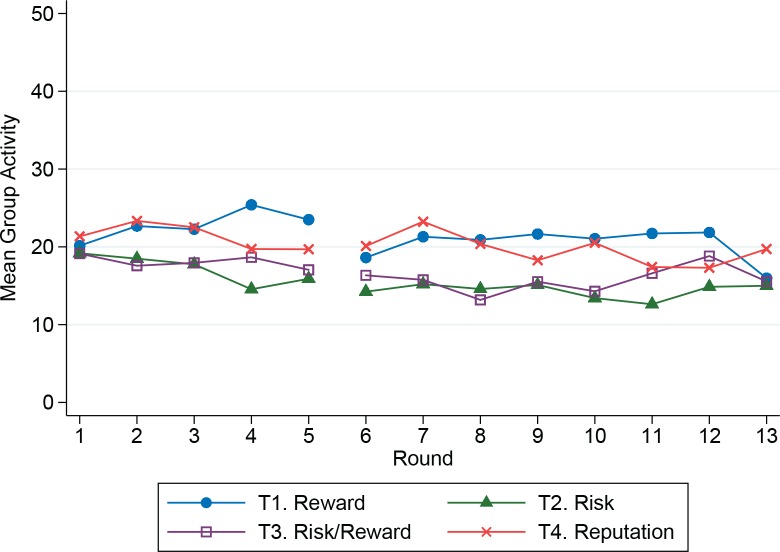
Mean allocation to the group activity.

Summary statistics are provided in Table 3, and Figs 1 and 2 present these means by treatment over time. Fig 1 clearly shows that for any given treatment, more resources were shared with those experiencing a shock. The regression analysis, discussed below, will show that this sharing was also conditioned on observed cooperative behavior. Although there appear to be differences among the treatments in the mean allocation to the group activity for periods 6–13, note that a similar pattern also holds for the Baseline in rounds 1–5 (Table 3 and Fig 2). In the regression analysis, after we control for the baseline level of cooperation, there were no significant differences among the treatments. Thus, although there were significant amounts of sharing, and this sharing was directed towards those who exhibit more cooperative behavior, this did not lead to an increase in individual allocations to the group activity.

**Table 3:** Summary Statistics.

	Mean Allocation to the Group Activity		Mean Sharing Amount Received (rounds 6–13)	
Treatment	Baseline (rounds 1–5)	Treatment (rounds 6–13)	Shocked	Not Shocked
T1-Reward	22.8 (12.8)	20.4 (15.2)	96.7 (71.3)	
T2-Risk	17.2 (13.0)	14.4 (12.0)	162.4 (96.2)	43.3 (40.3)
T3-Risk/Reward	18.1 (10.7)	15.8 (12.0)	153.4 (101.3)	50.7 (39.8)
T4-Reputation	21.3 (13.2)	19.6 (13.7)	259.0 (185.7)	48.6 (49.1)

Standard deviation in parenthesis.

### Sharing

In the Reward Treatment, the average amount received from sharing was 96.7 rubles. In the three treatments with idiosyncratic risk, sharing was predominantly directed toward those experiencing a hardship (Fig 2). Moreover, the more a shocked individual cooperated in the production domain, the more he or she received from sharing. We explore this result with four random effects regression models in Table 4, one regression for each of the four treatments with risk and/or rewards. The models all use the same basic structure: *Y*_*it*_ = β_0_ + β_1_ • θ_*it*_ + β_2_ •*t* + ω_*i*_ + ε_*it*_, where *Y*_*it*_ is the total amount received in sharing by subject *i* in round *t* ⊰ [6,13], θ_*it*_ is a set of independent variables that control for whether each individual was shocked, the amount shared in the previous period, the amount allocated to the group activity in the current period, and interactions of these variables, ω_*i*_ captures unobserved individual subject characteristics and ε_*it*_ represents the contemporaneous error term. Because subjects participated in multiple rounds of a single treatment, subject-specific heterogeneity is modeled as a random effect. We use a Huber [51] and White [52] robust estimate of variance. We report standard errors clustered at the group level.

**Table 4:** Individual Amount Received from Sharing (Rounds 6–13).

	**Reward**	**Risk**	**Risk /Reward**	**Reputation**
Amount Shared_*t*–1_	0.09 (0.06)	0.14 (0.12)	0.07 (0.06)	0.03 (0.07)
Group Activity_*t*_	1.15*** (0.39)	-0.31 (0.25)	-0.25 (0.36)	-0.22 (0.52)
Shocked_*t*_		108.24*** (24.53)	82.18** (32.99)	-40.70 (42.69)
Shocked_*t*_ X Amount Shared_*t*–1_		-0.14 (0.19)	-0.19 (0.13)	1.16** (0.55)
Shocked_*t*_ X Group Activity_*t*_		2.48 (1.51)	2.28** (1.14)	5.66*** (1.73)
Period_*t*_	-0.92 (1.43)	-1.18 (1.76)	-2.78** (1.16)	-2.30 (1.87)
Constant	72.71*** (22.64)	45.60** (22.27)	72.87*** (16.62)	73.13** (33.38)
N	280	161	210	161
R^2^	0.17	0.52	0.39	0.67

Robust standard errors are clustered at the group-level.

Statistical significance:

***: p<0.01

**: p<0.05.

Controlling for the subject characteristics described in Table 2 yields the same qualitative results.

The first model in Table 4 shows results for the Reward Treatment, which did not include a shock and therefore related variables are not included. Consistent with H1, the *Group_Activity*_*t*_ coefficient is positive and statistically significant. Conversely, whether the individual shared resources in the previous round was unknown and, as expected, the *Amount_Shared*_*t–1*_ variable is not significant. Thus, consistent with Sefton, Shupp, and Walker [48], individuals did use the sharing mechanism to reward cooperative behavior in the group activity decision. However, the magnitude of the effect was relatively modest. Each hour allocated to the group activity yielded an average return of 5.15 rubles—1.15 received from sharing plus 4 rubles from the group activity—which was only about half of the 10 ruble return from an hour allocated to the individual activity. Recall that every hour allocated to the group activity yields 4 rubles for the individual, as well as each of the other group members (20 rubles per hour which is evenly divided among all five group members).

**Fig 3:**
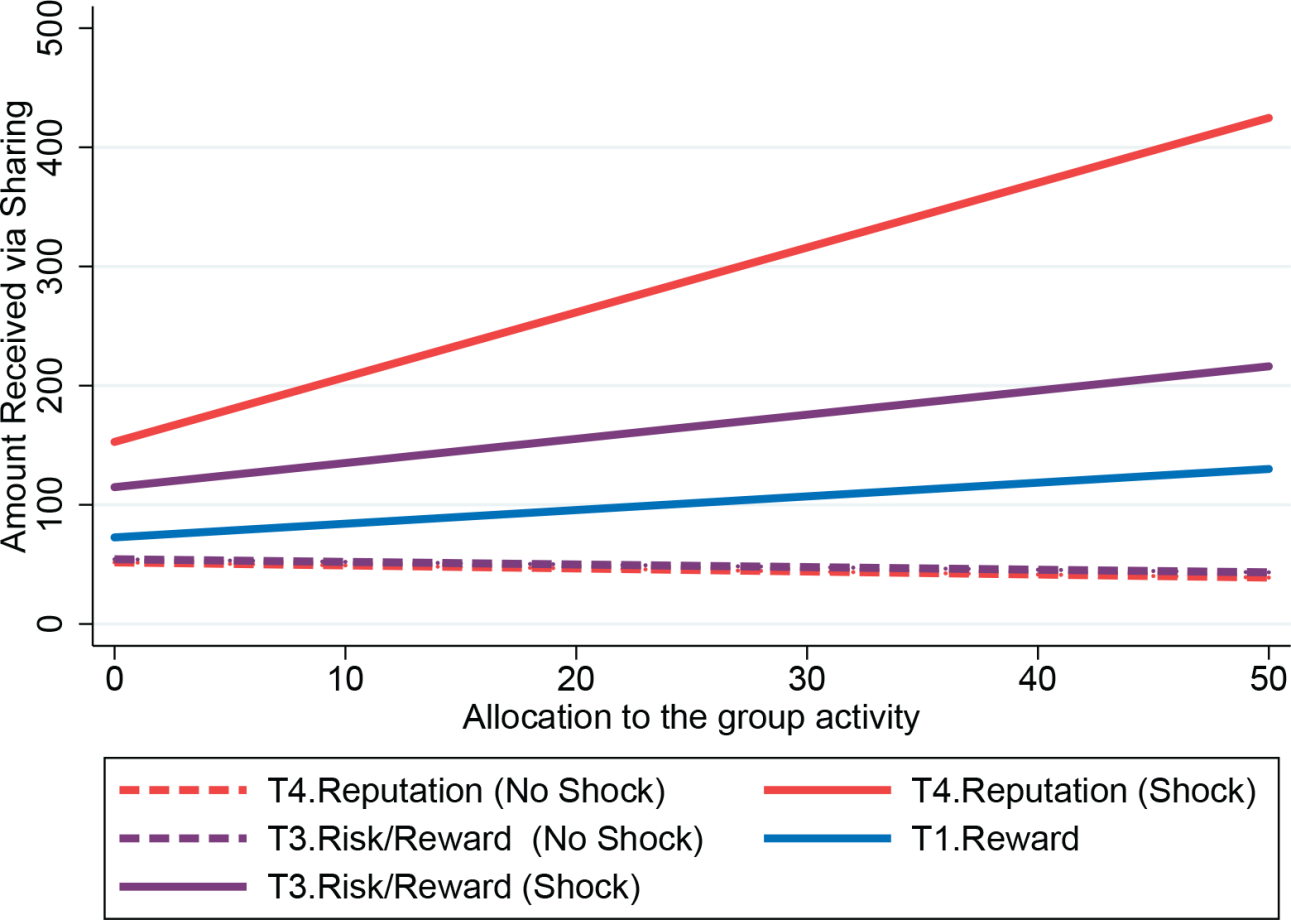
Predicted individual amount received in sharing, conditioned on whether the individual received a shock, using coefficients in Table 4.

The next three models include interactions of whether the individual was shocked, the allocation to the group activity in the current round, and the amount shared in the previous round. In all treatments, sharing decisions were affected by the available information, and unavailable information is not significant, as expected. In the Risk Treatment, participants received an average of 108.24 rubles whenever they incurred a shock, supporting the hypothesis (H2) that people used sharing to assist those in need. With more information about other group members’ behavior in the Risk/Reward and Reputation Treatments, sharing was still directed toward those in need, and the amount received increased for those individuals with higher levels of cooperation in the group activity (Fig 3). In the Risk/Reward treatment, those who experienced a shock continued to receive some support that was independent of their actions (82.18 rubles), but participants who incurred a shock also received an additional 2.28 rubles per hour allocated to the group activity (consistent with H3). In the Reputation treatment, behavior in both the current and previous rounds was common knowledge. Sharing was not used to reward cooperation independent of the shock (i.e. a Shocked coefficient of zero cannot be rejected). Instead, sharing was directed only toward those in need and was conditioned on their cooperation. In support of H4, sharing was conditioned on both the shocked individual’s most recent sharing decision (period *t*–1) and the most recent group activity decision (period *t*). For each hour allocated to the group activity, shocked players received 5.66 rubles from sharing. In addition, for each ruble shared in the previous period, shocked players received 1.16 rubles from sharing.

### Group Activity (Production Domain)

In the first five rounds, all groups participated in the Baseline Treatment. In the Baseline, average allocations to the group activity were about 40% of the initial endowment, consistent with results from other linear public goods games [2,53]. These allocations are lower than those reported from one-shot public goods games conducted previously in a neighboring region of Kamchatka [45]. Although that study was also conducted in Kamchatka, it is not directly comparable to ours due to differences in methodology, incentive structure, single versus multiple rounds, framing of the game and possible regional differences in cooperation. Table 5 presents the results of two random effects models for the group activity decision in each treatment with risk and/or reward (rounds 6–13 only). In these regressions the dependent variable *Y*_*it*_ is the individual allocation to the group activity of subject *i* in round *t*. We exploit the within-subject design by using the individual’s average group allocation over all five rounds of the Baseline Treatment as an independent variable (*Baseline Group Activity*). To test for differences among groups by treatment, we estimated a random effects model in which we regressed the allocation to the group activity in the Baseline treatment (rounds 1–5) with independent variables for stage 2 treatment and controls for subject characteristics (this model can be reproduced using the replication data referenced at the doi listed above). If all groups were identical, then the coefficients for stage 2 treatments should not be significant, but they are. Therefore, we control for these differences with the Baseline Group Activity variable.

**Table 5:** Individual Amount Allocated to Group Activity (Rounds 6–13).

	**Model 1**	**Model 2**
Reward Treatment	omitted	omitted
Risk Treatment	-1.642 (1.76)	-1.705 (1.66)
Risk/Reward Treatment	-0.961 (1.74)	-1.290 (1.82)
Reputation Treatment	0.380 (2.86)	0.361 (2.59)
Round	-0.095 (0.16)	-0.137 (0.17)
Baseline Group Activity	0.775*** (0.10)	0.752*** (0.10)
Male		-1.193 (1.50)
Age		0.108** (0.05)
Indigenous		-0.752 (2.08)
Education (years)		-0.232 (0.17)
Community 1		omitted
Community 2		-3.190** (1.45)
Community 3		-2.197 (1.74)
Constant	3.616 (2.70)	5.553 (4.13)
N	1088	1024
R^2^	0.20	0.23

Robust standard errors are clustered at the group-level.

Statistical significance:

***: p<0.01

**: p<0.05.

To test for potential omitted variable bias, Model 2 adds individual characteristics and fixed effects for the communities. We include as control variables gender, age, race, years of education and the community of origin; however, to protect participant confidentiality and to make data publically available for replication we have not identified specific communities or a specific ethnic group in the regression results. As indicated in Table 5, coefficients in Model 2 are consistent with results from Model 1. We do not find evidence that omitting gender, age, ethnic group, and community of origin biases our model estimates.

In both models, none of the treatments are statistically significant, and a test of whether they are jointly significant is also rejected (χ^2^ = 2.52, *p* = 0.47 for Model 2). Thus, contrary to H5, the ability to share failed to increase cooperation in both the Risk/Reward and Reputation treatments. Although results indicate sharing with those experiencing the shock was conditioned on the individual’s allocation to the group activity (Table 4), the levels of sharing were insufficient to induce an increase in cooperation. The expected return from allocating one hour to the group activity was approximately 4.3 rubles, which is about half of the expected return from an hour in the individual activity (8 rubles) and no different than the 4 ruble return from the group activity in the Baseline treatment. If shocked (with 20% probability), on average the individual would receive 5.66 rubles from sharing for each hour allocated to the group activity. If not shocked (80% probability), the individual would receive 4 rubles from the group activity. On the other hand, an hour allocated to the private activity yielded 0 if shocked (20% probability) and 10 if not shocked (80% probability).

Thus, we find some support for Panchanathan & Boyd’s [16] model of indirect reciprocity. Individuals in need did receive substantial support, and when possible, this support was conditioned on their reputations for cooperation. However, the benefits from a positive reputation did not exceed the costs of participating in the group activity in our experiment, and as a result, we find that the opportunity to share did not increase cooperation.

## Discussion

We systematically examined the interactions of strategic and environmental risks among people in Kamchatka who face these challenges repeatedly in the post-Soviet era [45]. Introducing idiosyncratic environmental risk in the social dilemma increased interdependence, and people responded by channeling resources to those in need, rewarding individuals for cooperation, and withholding support from individuals who did not cooperate. The ability to share as a tool to mitigate environmental risk increased the interdependence among group members. As a result, high levels of sharing were achieved without direct reciprocity or a strong commitment device. Observed sharing does, however, appear to reflect local sharing norms. We find strong evidence for sharing, even without reputations. This result is consistent with models of generosity driven by need-based transfers [54] as well as a model of pro-social behavior (and related experimental results) in which preferences for keeping social rules are the driving force behind pro-social behavior [55].

When current or past behavior was observable, sharing was conditioned on observed cooperative behavior. In the Reward Treatment, individuals who participated more in the group activity received more from sharing, consistent with previous studies that emphasize the importance of rewards, punishments, and reputations for the emergence of cooperation [50,56]. The positive relationship identified between sharing and allocations to the public good in the Risk/Reward and Reputation Treatments suggests that when both strategic and environmental risks are present in a social dilemma, the effects of strategic risks depend on environmental risks. These results have important implications for research on risk-pooling, the role of reputations, rewards, and punishments in theories of cooperation, and more generally, the role of environmental variability in human adaptation and resilience.

Ethnographic research on risk-pooling emphasizes the importance of supporting those in need and mechanisms of reputation to maintain cooperation [32,57]. Lab experiments inspired by this research have demonstrated that high-variance resources and reputations can play a key role in the emergence of risk-pooling, dramatically increasing reciprocal exchanges among individuals relative to low-variance resources [58] and that risk-pooling strategies can increase individual and pair-wise survival in environments with high degrees of risk [59,60]. Similarly, agent-based simulations have shown increased environmental harshness—which can be mitigated via cooperation—can amplify cooperation [61]. Each of these studies emphasizes the impact of interdependence on the emergence of cooperation. We contribute to this work by demonstrating how asymmetries of need caused by stochastic environmental risks or “shocks” interact with the strategic risks tied to rewards, punishments, and reputations to increase interdependence and enhance risk-pooling. In both the Risk/Reward and Reputation Treatments, individuals who contribute more to the public good receive more via sharing, but only when they suffer a shock. These interactions between strategic and environmental risks suggest strategic risks remain important for precisely those individuals who benefit most from risk-pooling, discouraging defectors and free-riders. Indeed, we found the effectiveness of risk-pooling increased when people had the ability to monitor and act upon reputations across multiple rounds. While previous research has emphasized the importance of exogenous commitment devices, formal institutions, endogenous group-formation, and direct reciprocity for effective risk-pooling, our experiments show that risk-pooling can emerge from endogenous reputation dynamics and indirect reciprocity.

Although the interaction of strategic and environmental risk enhanced the effectiveness of risk-pooling, we did not observe the systematic increases in contributions to the group activity reported by previous studies with rewards added to a social dilemma [8,48,50]. As indicated above, contributions to the group activity are no higher in the final two treatments with rewards than in Risk Treatment. One explanation is that the benefits of good reputations for cooperators never exceed the costs of contributing to the public good. Previous studies with a similar two-dilemma design amplify the impact of reputations by increasing the relative costs and benefits (i.e. efficiency) of rewards and/or punishments, often with ratios as high as 1:3 [3–5,8,62]. Thus, increasing levels of cooperation observed in previous experiments may not be due to the presence or absence of rewards and punishments *per se*, but the presence of *highly efficient* rewards and punishments [63–65]. While highly efficient reward/punishment mechanisms have been shown to increase levels of cooperation in experiments, it is less clear how often such mechanisms are available in naturally occurring contexts of cooperation [13]. Indeed, the way participants condition aid to needy players based on cooperation reflects local norms of indirect punishment, which are more commonly observed in our study region than norms of direct, costly punishment.

In addition to addressing individual strategic behavior, our study highlights the important role of factors that increase interdependence among individuals. We investigated one factor—stochastic resource acquisition—that increases interdependence by creating consumption deficits that can be overcome by pooling resources through sharing. Such deficits might also arise from differences in individual/household productive capacity and consumptive needs [66] or stochastic differences in harvests due to poor health or other misfortunes [67,68]. Our experiments incorporated production deficits via stochastic shocks, providing a specific factor for amplifying the impact of reputations relative to the highly efficient reward and punishment mechanisms utilized in previous studies.

Scholars studying processes of contemporary human adaptation to unprecedented forces of global climatic, economic, political, and cultural change have emphasized the crucial role of strategies that mitigate environmental risks [69]. Many components of contemporary adaptation—including the role of traditional ecological knowledge, social networks, institutions, and other forms of social capital—depend on cooperation among individuals to maintain resilience in the face of shocks and perturbations [70]. Therefore, understanding how environmental risks interact with strategic risks to affect the emergence and stability of cooperation can improve our attempts to adapt to the challenges we face in contemporary environments. Our research suggests theories of cooperation can contribute to this goal by investigating a broader range of factors that increase interdependence.
